# Differentiation of an Iranian resistance chickpea line to Ascochyta blight from a susceptible line using a functional SNP

**DOI:** 10.1186/s13568-022-01385-y

**Published:** 2022-04-16

**Authors:** Kiyanoush Zangene, Abbasali Emamjomeh, Farhad Shokouhifar, Mojtaba Mamarabadi, Nafiseh Mehdinezhad

**Affiliations:** 1 Department of Plant Breeding and Biotechnology, Faculty of Agriculture, University of Zabol, Zabol, Iran; 2 Department of Plant Breeding and Biotechnology (PBB), Faculty of Agriculture, University of Zabol, Zabol, Iran; 3 Research Center for Plant Sciences, Ferdowsi University of Mashhad, Mashhad, Iran; 4 Department of Plant Protection, Faculty of Agriculture, Ferdowsi University of Mashhad, Mashhad, Iran

**Keywords:** Cell surface receptors, Kinase domain, LRR-based cell surface interaction network, Marker assisted selection

## Abstract

Identification of resistant sources to Ascochyta blight (AB) has been considered as a main purpose in most chickpea breeding programs. Achievements to molecular markers related to resistance to *Ascochyta rabiei* allows selection programs to be developed more accurately and efficiently. The aim of this study was to investigate the applicability of a functional SNP in differentiating Iranian resistant cultivars to be used in selection programs. Amplification of SNP-containing fragment with specific primer pair and its sequencing resulted in tracking and determining the allelic pattern of SNP18, SNP18-2147, SNP18-2491 and SNP18-2554 loci belong to *GSH118* gene in ILC263 (sensitive) and MCC133 (resistant) chickpea lines. Mutations in SNP18 and SNP18-2147 occur at the protein level at positions 499 and 554. Bioinformatics studies have shown that the *GSH118* gene is a Lucien-rich repeat receptor kinases (LRR-RKs) and encodes a membrane protein which can be involved in recognizing microorganisms and initiating immune signaling pathways in plants. Additional studies to determine the function of this gene and its interaction with other proteins can be effective in gaining more knowledge about the molecular basis of resistance against AB.

## Introduction

Chickpea farming with special importance in the period of soil rotation and fertility has always been considered in most parts of the world. Nutritional value and the rich plant protein of the seeds of this plant also play a special role in human diet (Jukanti et al. 2012). Chickpea cultivation in the western provinces of Iran is very important, especially in crop rotation in dry land crops (Naderi et al. 2013). Chickpeas in Iran with a harvest area of 5900 hectares of irrigated crops and 496 hectares of rain-fed crops are ranked fourth in the world, after India, Australia and Pakistan. However, Iran in terms of chickpea production is ranked eighth in the world, in terms of average yield in irrigated agriculture is ranked seventeenth and in terms of average yield of rain-fed cultivation is ranked 50th in the world (FAO 2016). Therefore, water constraints should be overcome as much as possible to increase yield and production of this crop. Attempts to make more use of winter rainfall have focused on the autumn cultivation of this plant (Tayyar et al. 2008; Sadeghipour and Aghaei 2012; Porsa et al. 2016). However, high humidity and cool temperature increase the possibility of Ascochyta blight (AB) disease and cause a lot of damage to the aerial parts of chickpea plant (Özdemir and Karadavut 2004; Peever et al. 2012). The damage of this disease is very serious in some rainy years in Iran and it can destroy 90% of this crop. Although comprehensive information on the damage of this disease is not available in consecutive years however, according to the report of Kermanshah Agricultural Organization, in 2018 in some region of Kermanshah province in western Iran, on a scale of about 6,200 hectares of land under chickpea cultivation, AB has caused 90% damage to the chickpea fields which was due to intermittent rainfall and high humidity so that, the farmers in this area have sustained a loss around 1400 billion IRR (Iranian Rial) in that year (Retrieved from the website of Young journalists club on May 23, 2018, https://www.yjc.news/00RRzt).

Genetic diversity and pathogenicity of the isolates of this fungus have been studied in different regions of Iran and the reports showed high diversity of this pathogen in different regions (Farahani et al. 2019, 2021; Nourollahi et al. 2011; Shokouhifar et al. 2003a, b). A comprehensive study performed on the collected Ascochyta isolates from different regions of Iran has shown that six pathotype groups of *A. rabiei* are distributed in western, northwestern, central and northeastern regions of Iran (Shokouhifar et al. 2003b). The study on the mating type of isolates in different regions of the country confirms the presence of both reproductive types in different regions of Iran (Farahani et al. 2021; Mahmoodi and Banihashemi 2004; Nourollahi et al. 2011). The presence of high diversity and reproductive types in the population of this pathogen indicates that if favorable environmental conditions are provided, there is a possibility of serious damage to the chickpea cultivation in different parts of Iran.

Cultivation of resistant cultivars of chickpea against AB has been considered as an economic solution but due to the genetic diversity and high pathogenicity in the population of this fungus (Shokouhifar et al. 2003b; Hosseinzadeh Colagar and Barzegar 2008; Poorali Baba et al. 2008) identification of sustainable resistance sources has been considered as a main goal in most chickpea breeding programs (Shokouhifar et al. 2006; Kanouni et al. 2011; Newman et al. 2021). Based on the study of genetic diversity of global chickpea germplasms using sequencing methods, it has been determined that Iran is one of the main routes for chickpea spread from Turkey to North Africa and India (Varshney et al. 2021). It has also been shown that many sources of resistance to AB such as ICC3996, ICC14903 and ICC13729 are of Iranian ancestry (Li et al. 2017). Numerous studies have been performed to evaluate the germplasm of Iranian chickpeas (Farahani et al. 2019; Vafaei et al. 2017). Identification of four resistant lines against six pathotypes of *A. rabie* during field and greenhouse studies on different chickpea germplasm provided by Seed Bank of Research Center for Plant Sciences, Ferdowsi University of Mashhad, Iran confirmed the richness of Iranian genetic resources (Shokouhifar et al. 2006). Therefore, it can be expected that the identification of resistance sources to this pathogen will be facilitated using new methods, and it will be possible to evaluate more chickpea lines and cultivars.

Several QTL have been reported on eight linkage groups (LG1-LG8) that could explained majority of AB resistance in chickpea genome (Sharma and Ghosh 2016). LG4 have been frequently mentioned in several reports as good AB resistance candidate source (Sudheesh et al. 2021). Some efforts aimed to identify AB resistance related genes arrested in LG4 genome area using transcriptional profiling differentially expressed genes in AB infected chickpea resistance and susceptible lines in comparison with uninfected plants (Leo et al. 2016; Madrid et al. 2013).

According to the numerous information about molecular markers and the linkage maps showing chickpea genome regions associated with resistance to AB (Deokar et al. 2019) and also chickpea genome project data (Varshney et al. 2013) and sequencing data obtained from chickpea genome and transcriptome to identify the differences among resistant cultivars using modern techniques (Li et al. 2017; Deokar et al. 2019; Garg et al. 2019; Maurya et al. 2020) all have made this subject possible to identify the resistance related genes that can be used as markers to select sources of resistance against AB.

Li et al. (2017) have studied 59 chickpea genotypes with different degrees of resistance to AB using Whole Genome Re-Sequencing (WGRS) technique and they identified over 800,000 SNPs. The Genome-Wide Association Studies (GWAS) showed that a 100 Kb region AB4.1 located on the chromosome No.4 of chickpea is associated with resistance to AB and described about 22% of resistance to AB. Twenty one resistance related point mutations were identified in this region among which they introduced a functional SNP (Ca4:15.920.939) which located in the gene Ca05515, that could be considered for differentiation of resistant and susceptible cultivars from each other (Li et al. 2017). Also they analyzed expression of the gene Ca5515 in 6 resistance and susceptible chickpea lines 24 and 48 h after inoculation with *A. rabiei* and showed the expression was significantly up regulated in two of three resistance lines, which indicating this gene may not be responsible for the AB resistant or maybe one of the chickpea lines have different resistance mechanisms against *A. rabiei*. So they concluded this information could not be used into screening programs before evaluation by phenotyping technology.

In another study, ten RGA genes (Resistance Gene Analog) were selected based on the genomic data retrieved from the gene bank and their expression pattern was quantitatively investigated in resistant and susceptible chickpea plants at different hours after AB infection. The results showed that, the expression of four genes were significantly increased right after inoculation, during spore germination and the beginning of penetration into the plant’s epidermal tissues in the resistant genotype ICC3996 (Zhou et al. 2019). Finally, they have concluded that, the decision to use these genes in breeding programs depends on evaluating their association with resistance to AB during complementary experiments.

Indeed further research is warranted to indicate correlation of the SNP with the resistance level of more Chickpea lines and analysis their linkage in segregated F2 population derived from crossing between the resistance and susceptible lines.

The aim of this study was to validate the applicability of the functional SNP (Ca4:15.920.939) for differentiation of an Iranian resistant line that was selected from the germplasm of Iranian chickpea against 6 pathotypes of AB disease, in order to be used as molecular marker for evaluation of the samples retained in the germplasm of Iranian chickpea seed bank and the F2 progeny of the segregating population resulted from crossing the resistant and susceptible chickpea lines. Tracking the SNP and the region surrounding it led to the detection of new mutations. In this study, the gene associated with this SNP was analyzed and the location of mutations in the functional domains of the gene was investigated.

## Material and methods

Two chickpea lines named ILC 263 and MCC 133 were prepared from a private seed bank belongs to the Research Center for Plant Sciences, Ferdowsi University of Mashhad, Iran. They were introduced as sensitive and resistant to AB in the previous studies (Shokouhifar et al. 2003a, 2006), respectively. Pathotype III of *Ascochyta rabiei* (Shokouhifar et al. 2003a) was provided by the Microorganisms Collection of Ferdowsi University of Mashhad (WDCM 1207), Iran. Five seeds were planted in each pot from each line to confirm the resistance level of seedlings related to ILC 263 and MCC 133 lines. The seeds belonging to MCC133 line were obtained from a single resistance seedling selected in a previous studies (Shokouhifar et al. 2006). After germination, three seedlings related to each line were preserved and healthy and young leaves were sampled for DNA extraction using a method suggested by Doyle and Doyle (1987). The stage of plant infection was performed according to the methods recommended by different literature (Shokouhifar et al. 2003a; Fondevilla et al. 2015; Bayraktar et al. 2016) with minor modifications. In this method, seedlings were inoculated at 4–5 leaf stage with suspension of mycelium and spores collected from a 20 days old fungus grown on a Potato Dextrose Agar (PDA) plate with 9 cm diameter. The inoculated plants were kept under plastic cover for 24 h in dark condition at 22 °C temperature and 85% relative humidity. The plants were then transferred to the growth cabinet for 72 h under 16 h of light and 8 h of darkness with 85% relative humidity. They were transferred to a greenhouse with average temperature of 16–22 °C. Disease symptoms and imaging were performed daily for 14 days after inoculation. The disease symptoms was scored based on a nine point index (Singh et al. 1981), where 0.0–1.0 = no visible disease symptom on any plant; 1.1–3.0 = disease lesions visible on less than 10% of the plants, no stem girdling; 3.1–5.0 = lesions visible on up to 25% of the plants, stem girdling on less than 10% plants but little damage; 5.1–7.0 = lesions present on most of the plants, stem girdling on 50% of plants; 7.1–9.0 = lesions coalesced on plants, stem girdling present in more than 50% of plants. Based on the disease severity score, accessions were categorized for their reaction to AB infection as follows: 0.0–1.0 = asymptomatic or highly resistant (HR); 1.1–3.0 = resistant (R); 3.1–5.0 = moderately resistant (MR); 5.1–7.0 = susceptible (S); and 7.1–9.0 = highly susceptible (HS).

Retrieval of the functional SNP containing sequence reported by Li et al. (2017) was performed by searching for the SNP-containing sequence in the chickpea genome sequence with following accession number GCA_000331145.1 using the Genome Data Viewer software (https://www.ncbi.nlm.nih.gov/genome/gdv), which have been computationally predicted with Identification Number 105851082. The schematic view of the gene, its features and the position of SNP in the gene besides its encoded protein were defined using SnapGene V1 software. Specific primers containing target SNP were designed using Prime premier V3 software and synthesized by Sinaclon Company (Tehran, Iran).

Tracking and amplification of the fragment containing target SNP in genomic DNA extracted from ILC263 and MCC133 chickpea lines were accomplished using PSh118.2-F (5′-GAC TGG ACC ACA AGG CTG AAGA-3′) and PSh118.2-R (5′-AGT ACC ACC ACA AGC ATC TTC AGA-3′) primer pairs. PCR was performed in a thermocycler (Eppendorf, Germany). Each PCR reaction was included 5 μl MasterMix 2X (Amplicone co. Brighton, UK), 5 Picomoles of each specific PSh118.2-F/R primers, 1 μl genomic DNA (~ 50 ng) and required deionized distilled water up to 10 μl. PCR protocol was 3 min at 93 °C, then 35 cycles of 45 s at 92 °C, 40 s at 60 °C and 1 min at 72 °C, followed by a final extension for 5 min at 72 °C. The amplification product was analyzed by electrophoresis on 1% agarose gel and stained by DNA green viewer and visualized under UV light with a gel analysis system (UviDoc, UK). The PCR products were purified using PCR Purification Kit (BioNEER, South Korea) following the procedure described by the manufacturer. Specific amplified fragments related to each chickpea cultivar were sequenced bi-directionally using PSh118.2-F and PSh118.2-R primers by Sinaclon Company (Tehran, Iran). The quality of the sequencing results was evaluated using DNA Baser V4 software and the approved regions from the two directions of the sequences were assembled for each fragment. Alignment of sequencing results of each line in comparison with the reference sequence of Gene ID: 105851082 was performed using CLC Genome Workbench software and nucleotide diversity was identified afterwards. The nucleotide sequence of the amplified region related to the different chickpea lines was translated into amino acid sequence and their alignment with the reference sequence was performed in CLC Genome Workbench software in order to determine the protein locus of the mutations.

Identification of homologous proteins in contiguous species was performed using BlastP tool in the reference protein bank. The proteins with above 95% overlapping and more than 60% identity were selected as the highest amount of affinity and their phylogenetic tree was plotted using the Gene tree link at NCBI. Protein domain sequences related to the considered gene were identified using the SMART database and the conserved domains was recognized through the related links. The active loci of the domains were identified in the NCBI database and the position of the identified SNPs and the target SNP were compared with the active locus of the domains. The location of the protein in the cell membrane was investigated using a Protter web base tool (https://wlab.ethz.ch/protter) and the effects of mutations on protein structure were studied using SnpEffec V4 (https://snpeffect.switchlab.org).

## Results

**Fig. 1:**
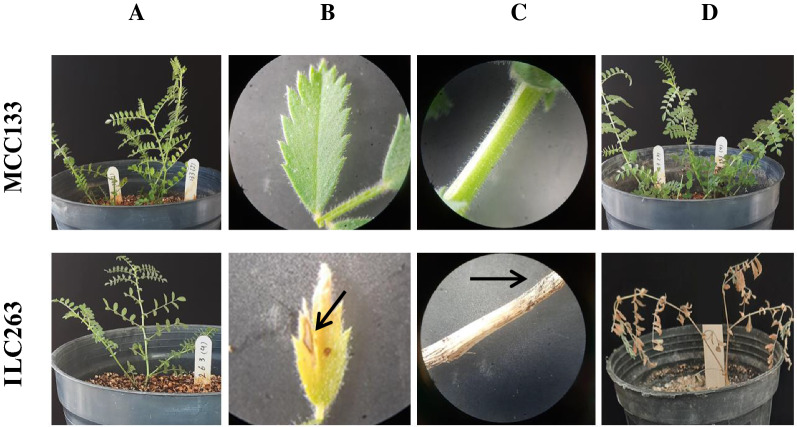
The damage of *Ascochyta rabiei* Pathotype III on ILC263 (sensitive) and MCC133 (resistant) chickpea lines. **A** (Plants just before inoculation), **B** and **C** (Disease symptoms on the leaves and stems 15 days after inoculation with 10× magnification) and **D** (Inoculated plants 15 days after inoculation). The arrows are shown the disease symptoms on the inoculated leaves and stems

The pathogenicity test of *Ascochyta rabiei* Patotype III was performed on the seedling of ILC263 (sensitive) and MCC133 (resistant) lines in the stage three to four leaves in order to confirm the resistance level of the evaluated lines. The first symptoms of AB was appeared on the sensitive line (ILC263) 7 days after inoculation (Fig. 1B). The disease symptoms were gradually developed on the leaves and stems and the plants were completely damaged until the fourteenth day after inoculation and the damage score equal to 9 was recorded on chickpea plant which means the complete death of the plant was occurred (Fig. 1D). At the same time in the resistant line (MCC133) no symptoms of disease appeared and the damage score equal to one was recorded on inoculated plants (Fig. 1A–D).

Mutation No. 18 was selected amongst 21 point mutations associated with resistance to AB. This mutation had already been reported as a functional mutation by Li et al. (2017). The DNA sequence containing this mutation was tracked within the genomic data of chickpea in NCBI genomic browser in order to determine its pattern in Iranian chickpea line resistant to AB (Shokouhifar et al. 2006). The mutation was named SNP18 and it was located within the predicted gene sequence with following Gene ID: 105851082. The length of this gene is 2662 bp and it carries two exons with lengths of 1318 and 665 bp which are separated by an intron with 679 bp length. Based on Gene Bank software prediction, this gene encodes a hypothetical protein which comprises 660 amino acids and has 72.5 kDa weight. This gene was temporarily named *GSH118* up to functionally be analyzed. The Blast of this protein against chickpea translated short read and non-redundant protein database of NCBI with e-value cut-off ≤ 1e−05 and similarity ≥ 40% showed no more hit at the time of this report.

**Fig. 2:**
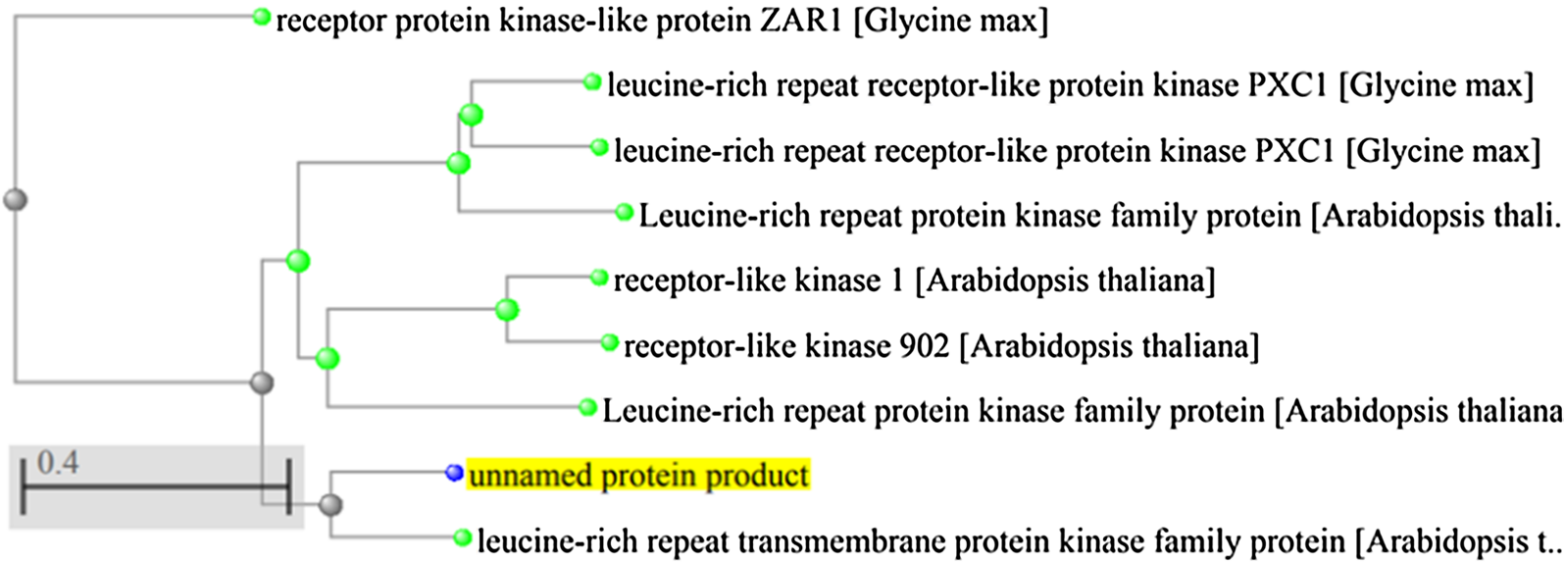
The results of GSH118 protein sequence searched in the reference protein database with over 95% overlapping, the phylogenic tree was constructed using a method based on fast minimum evolution with a maximum sequence difference of 0.85 using the Grishin distance model

However, the blast results against the NCBI reference protein database showed that homologs of this protein are present in other plant species, including Arabidopsis and soybean, with over 95% overlapping and more than 45% identity. A membrane protein with accession number NP_177007 and length of 670 amino acids related to Arabidopsis showed the highest affinity among them (Fig. 2).

**Fig. 3:**
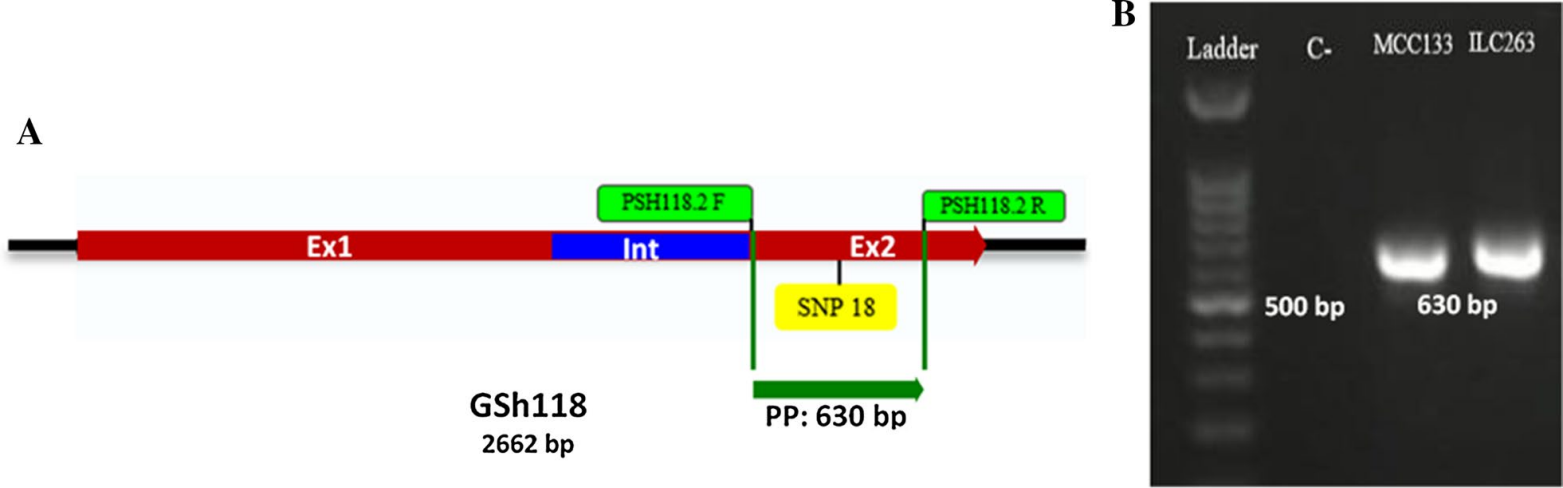
Schematic representation of *GSH118* gene showing the positions of primers and SNP (**A**), electrophoretic pattern of amplified fragments from the genomic DNA of MCC133 and ILC263 chickpea lines using PSh118.2-F/R primer pair (**B**). Ex1, EX2 and Int are first exon, second exon and intron, respectively. PP: PCR product, Ladder: 100 bp DNA size marker, C^−^: negative control

The positioning of SNP18 sequence showed that this mutation was located on the second exon in the position number of 2349 from the start codon and located in the middle nucleotide of the codon number 557 (Fig. 3A). The presence of *GSH118* gene in chickpea lines was confirmed using PSh118.2-F/R primer pair which have been designed in the appropriate distance upstream and downstream of SNP18 (Fig. 3A). The PCR results showed that specific single bands could be amplified to the expected size of 630 bp (Fig. 3B). The electrophoretic pattern showed that the desired fragment was present in both MCC133 (resistant) and ILC263 (sensitive) lines and the designed specific primers amplified a unique band in both lines.

**Fig. 4:**
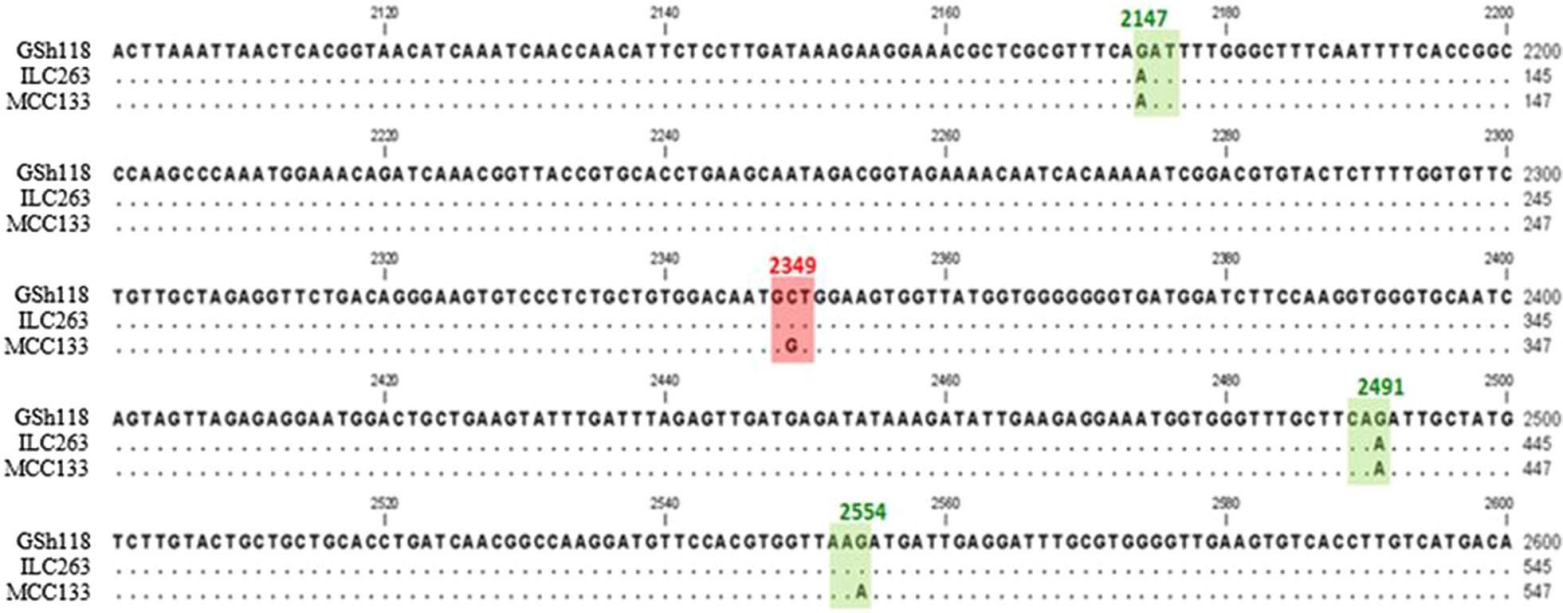
Multiple sequence alignment of the DNA region amplified by PSh118-F/R primer pair in two chickpea lines; MCC133 (resistant) and ILC263 (sensitive) in comparison with the reference sequence presented in gene bank (Gene ID: 105851082). Numbers are the position of the nucleotides relative to the start codon. Dark backgrounds show the position of SNPs in the codon. The 2349 position is corresponded to SNP18. The positions of 2147, 2491 and 2554 are related to SNP18-2147, SNP18-2491 and SNP18-2554, respectively. Similar sequences are shown as dot

The amplified fragments were sequenced in two directions in order to determine the nucleotide sequence at SNP18 position in resistant and sensitive lines. Multiple sequence alignment of sequencing results with sequence of *GSH118* gene showed that at the position of SNP18, the sequence of resistant line is different from the reference gene and sensitive line. In this position, the nucleotide of Guanine was observed in resistant line, while in the susceptible line, similar to the reference sequence, the nucleotide of Cytosine was observed at the same position (Fig. 4).

Alignment of the amplified sequences with reference sequence resulted in the identification of three new SNPs at 2147, 2491 and 2554 positions where all located in the coding region and named SNP18-2147, SNP18-2491 and SNP18-2554, respectively. More observations showed that unlike to SNP18, which is located in the middle position of the codon, SNP18-2147 is presented in the first position of the codon, and SNP18-2491 and SNP18-2554 are placed in the third position of the codon (Fig. 4).

The nucleotides presence pattern at SNP18-2147 and SNP18-2491 positions were the same in both resistant and sensitive lines but different from the reported reference sequence. The results showed that at SNP18 and SNP18-2554 positions, the nucleotide sequences are different between resistant and sensitive lines, and based on this, the resistant line can be differentiate from the sensitive line (Fig. 4).

**Fig. 5:**
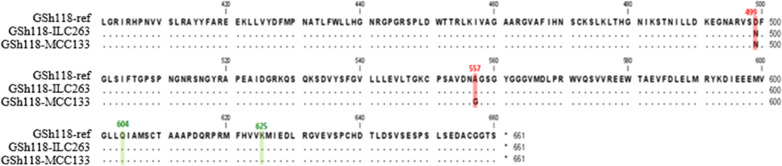
Regional protein sequence alignment comprising the identified SNPs of two chickpea lines; MCC133 (resistant) and ILC263 (sensitive) in comparison with the protein sequence predicted in the gene bank (XP_012570257.1). Numbers on the sequences are the amino acid positions from the beginning of the protein. Dark backgrounds show the position of SNPs in the codon. The positions 499 and 557 are corresponded to SNP18-2147 and SNP18, expressed at the protein level. The positions 604 and 625 related to SNP18-2491 and SNP18-2554 are not expressed at the protein level. Similar sequences are shown as dot

Peptide sequence alignment of amplified fragments related to MCC133 and ILC263 lines with putative and undescribed protein sequences encoded by *GSH118* gene showed that the variation observes only in the position of two amino acids (Fig. 5). More investigation on the positions of SNP18 showed that the amino acid located on the position of 557 substitutes under the influence of point mutation, so that, in this position, the amino acid of Alanine is present in the reference sequence and also the sequence of sensitive line (ILC263), while in the resistant line (MCC133), the amino acid of Glycine occurs due to a point mutation.

Among the three newly identified mutations, only SNP18-2147 was non-synonym and changed the amino acid at the position of 499 in the protein sequence. At the same position, the amino acid of Aspartate encoded by the *GSH118* gene is expressed in the hypothetical protein sequence, while in the MCC133 and ILC263 lines the Asparagine amino acid is similarly encoded (Fig. 5).

The amino acids presented on 604 and 625 positions were not affected by SNP18-2491 and SNP18-2554 mutations so that, in the sequence of the studied chickpea lines and the putative protein sequence encoded by *GSH118* gene, the amino acids of Glutamine and Lysine are observed, respectively (Fig. 5).

Genotype determination of MCC133 (resistant) and ILC263 (sensitive) lines based on four SNPs at the level of nucleotides and amino acids, showed that resistant and sensitive MCC133 and ILC263 lines can be differentiated from each other based on SNP18 at the DNA level (Table 1). In the other three positions, although the studied lines are different from the reference sequence, they are the same. Despite the fact mutations at the protein level occur at the SNP18 and SNP2174 positions, only change in the amino acid of 557 makes it possible to distinguish between the MCC133 and ILC263 resistant and sensitive lines (Table 1). Based on these results, it can be expected that the *GSH118* gene is probably a candidate gene related to the resistance pathway against *Ascochyta rabiei* in chickpea.

**Table 1:** Genotypes of MCC133 (resistant) and ILC263 (susceptible) lines in the position of SNP18 and three newly identified SNPs, showing the position of the SNP within the codon in the DNA sequence, relative to the start codon in the putative *GSH118* gene (GeneID: 105851082), and the position of the encoded amino acid and the presence or absence of point mutations at the protein level

SNP name	Position (N/P)	Ref. Seq	ILC263(S)	MCC133(R)
SNP18	2349	G*C*T	G*C*T	G*g*T
	557	Alanine	Alanine	Glycine
SNP2174	2147	*G*AT	*a*AT	*a*AT
	499	Aspartate	Asparagine	Asparagine
SNP2491	2491	CA*G*	CA*a*	CA*a*
	604	Glutamine	Glutamine	Glutamine
SNP2554	2554	*G*AT	*a*AT	*a*AT
	625	Lysine	Lysine	Lysine

The italic letters show the position of point mutations in their associated codons

**Fig. 6:**
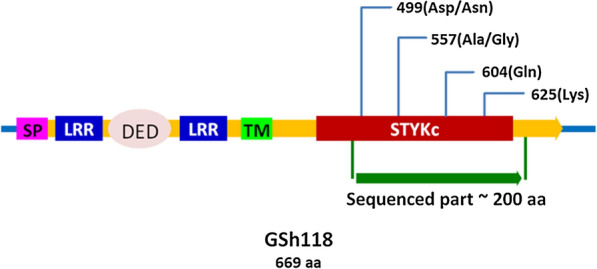
Schematic representation of the putative protein encoded by the *GSH118* gene, showing the domains and positions of SNPs identified in the sequencing region of MCC133 and ILC263 lines. SP: Signal Peptide domain, LRR: Lucien Rich Repeated domain, DED: Death-Effector Domain (apoptosis related domain), TM: Trans-Membrane domain and STYKc: Serine/Threonine/Tyrosine Kinases protein kinase domain

In order to investigate the effects of four following mutations; *GSH118*, SNP18-2147, SNP18-2491 and SNP18-2554 on the structure and function of the putative protein encoded by the *GSH118* gene, first the sequence analysis of this putative protein was performed in SMART and NCBI databases. The results showed that several important domains are present in the sequence of this protein (Fig. 6). Moreover, the results of this analysis showed that this protein belongs to a large family of proteins called leucine-rich repeat receptor-like protein kinase and has the Serine/Threonine Kinases (STKs) and Interleukin-1 Receptor Associated Kinases (IRAKs) domains. This protein is a membrane protein with Signal Peptide (SP) and Trans-Membrane domain (TM) and has several LRR domains in its N-terminal. A Death-Effector Domain (DED) is also present in this protein (Fig. 6).

**Fig. 7:**
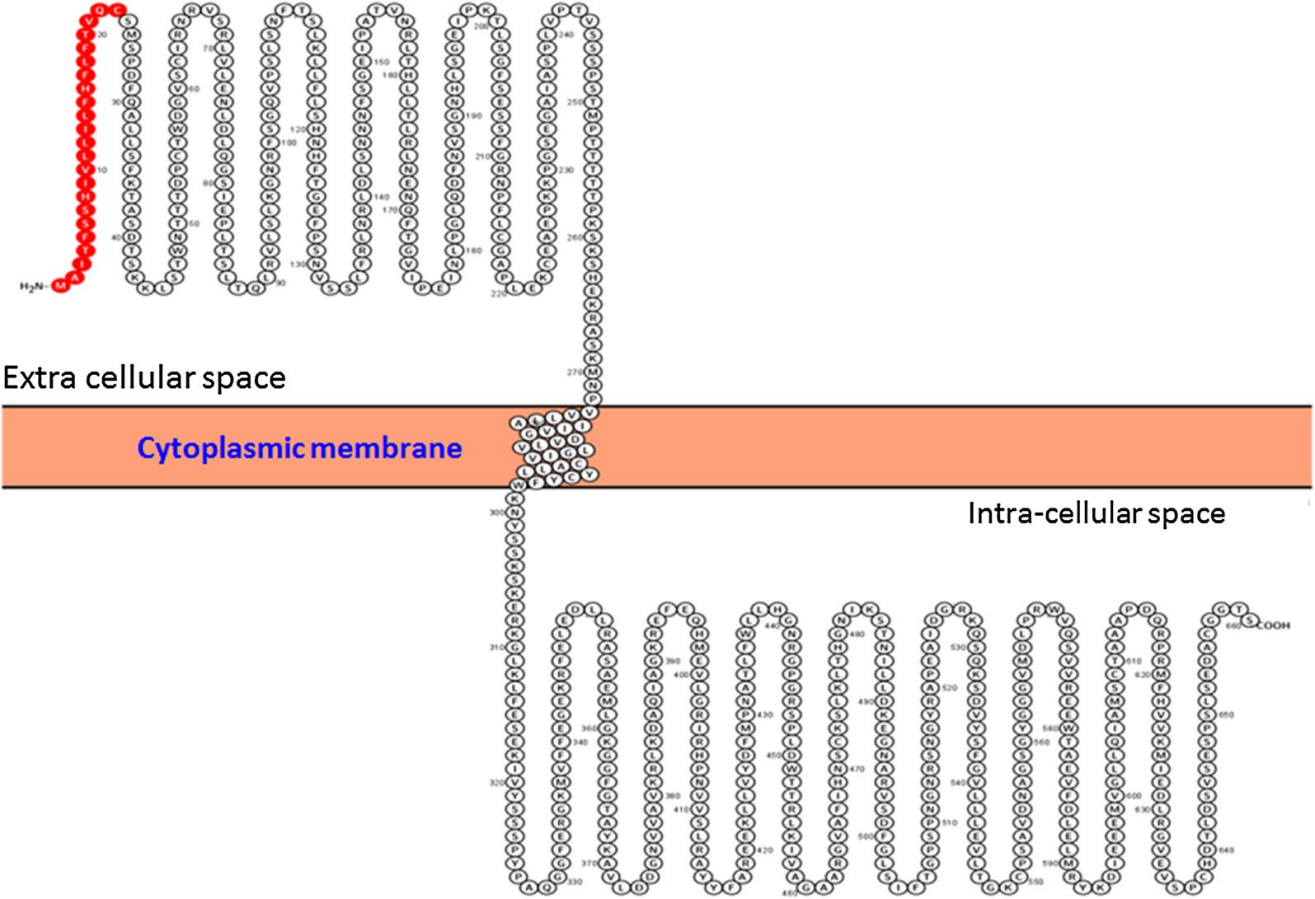
Schematic representation of the putative protein encoded by the *GSH118* gene relative to the cytoplasmic membrane predicted by Protter web tool. The circles are amino acids and numbers show the amino acids position. The red circles are signal peptide’s amino acids

**Fig. 8:**
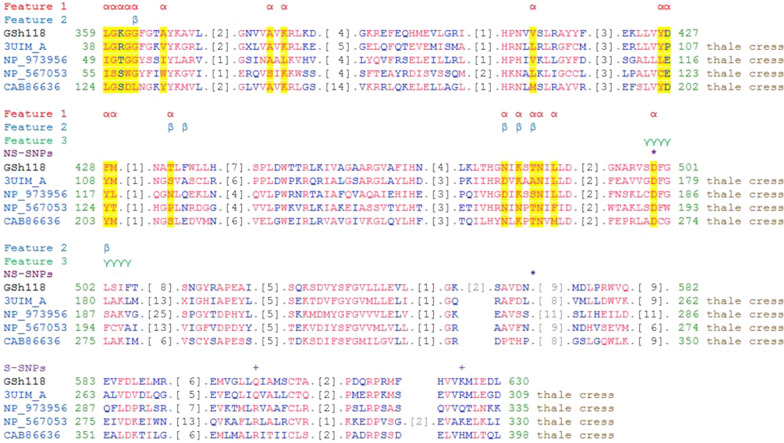
Multiple amino acid sequence alignment of STKs and IRAKs domains in the putative protein encoded by *GSH118* gene in the sequencing region of MCC133 and ILC263 lines compared to the domains (3UIM_A, NP_973956, NP_567053 abd CAB86636) reported in *Arabidopsis thaliana* (thale cress). α: Feature 1 (ATP binding conserved residues), β: Feature 2 (Polypeptide substrate binding site conserved residues), γ: Feature 3 (Activation loop conserved residues), *: Non-synonym SNP (NS-SNP) and + : Synonym SNP (S-SNP)

Sequence analysis of the putative protein encoded by *GSH118* in the Protter web tool (https://wlab.ethz.ch/protter) showed the presence of a signal peptide sequence at the amino terminus of the protein which is responsible for secreting of protein out of the cytoplasmic matrix. Next to this amino terminus, there are several leucine-rich domains (LRRs) from the position 24–272 which are located in the extracellular space. The domain of cytoplasmic membrane is situated in the range of amino acids 273–298 and, like an anchor, causes the protein to be stablished in the cytoplasmic membrane. At the carboxyl end of the protein, its catalytic domain, along with several other LRR domains, are located within intracellular space (Fig. 7).

the STKs domain catalyzes the transfer of gamma phosphorus from ATP to the amino acids Serine and Threonine, and IRAKs domain is involved in the Toll-Like Receptor (TLR) and interleukin-1 (IL-1) signaling pathways and plays a key role in innate immune responses. Comparing the corresponding sequence of GSH118 protein with the conserved domain features in the NCBI database showed that There are several active sites in these domains such as ATP binding conserved residues (α), polypeptide substrate binding site conserved residues (β) and activation loop (A-loop) conserved residues (γ), and also there are conserved amino acids in each of these sites (Fig. 8). More investigation on the position of the desired SNPs showed that SNP18-2147 in the amino acid position of 499 is located at ATP binding conserved residues (α) and activation loop conserved residues (γ). At this position of the STKs domain, the amino acid of Aspartate is conserved in the reference proteins of the Arabidopsis (3UIM_A, NP_973956, NP_567053 and CAB86636) while the amino acid of Asparagine has been observed in the sequence of MCC133 and ILC263 lines at the same position (Fig. 8).

Given the similarity of both amino acids i.e., Aspartate and Asparagine in terms of hydrophobicity and polarity, the only difference is related to the addition of the amino group to Asparagine which can only change its pH. Accordingly, the D499N mutation can be effective in fungal-plant interactions and pH-induced changes.

Analysis of the D499N and A554G mutations in SnpEffec V4 web based software (https://snpeffect.switchlab.org) showed that these mutations had no effect on protein aggregation tendency. In case of change in aggregation tendency, protein folding can be affected and its performance will change consequently.

## Discussions

The aim of this study was to select a functional marker to differentiate Iranian resistant and susceptible chickpea lines from each other in order to be used in the evaluation of the deposited samples in the germplasm of Iranian chickpea seed bank. For this purpose, a functional SNP was selected based on the results reported by Li et al. (2017). Moreover, based on the results of selection programs implemented by Shokouhifar et al. (2006), and Shokouhifar et al. (2003a) the lines of MCC133 and ILC263 were selected as a resistant and susceptible lines to AB, respectively. In the beginning, the resistance level of the evaluated lines was determined on the seedlings by pathogenicity test using *Ascochyta rabiei* (Patotype III) under standard conditions (Shokouhifar et al. 2003a). The degree of damages on these lines against *Ascochyta rabiei* pathotypes was consistent with the results of our previous studies (Shokouhifar et al. 2003a, 2006). Therefore, these results confirmed the resistance level of the evaluated seedlings from MCC133 line once again. The disease symptoms and damages on the sensitive line (ILC263) confirmed the pathogenicity of the fungal isolate related to Pathotype III used in this study and also showed that the conditions for inoculum preparation was achieved as well. Moreover, these results showed that the moisture conditions after inoculation were suitable for the onset of symptoms. Accordingly, sampling of seedlings related to MCC133 and ILC263 lines can be used as resistant and sensitive plants for molecular tracking of markers and genes related to resistance against AB.

In the recent study the presence of *GSH118* gene was confirmed using PSh118.2-F/R primer pair by PCR amplification of a 630 bp fragment in both MCC133 (Iranian resistant) and ILC263 (International sensitive) lines. Sequencing of amplified fragments and their alignment with the reference sequence resulted in the identification of two nucleotide alleles (G and C) at the position of SNP18. The alignment results also led to the identification of three new mutations at the positions of 2147, 2491 and 2554, all of which were located in the coding region and named SNP18-2147, SNP18-2491 and SNP18-2554, respectively. SNP18 was located in the middle position of the codon, SNP18-2147 was placed in the beginning position of the codon, and SNP18-2491 and SNP18-2554 were presented in the third position of the codon. Based on the results, by determining the nucleotide sequence at SNP18 and SNP18-2554 positions, the MCC133 resistant line can be distinguished from the sensitive ILC263 line. The loci of SNP18, SNP18-2147, SNP18-2491 and SNP18-2554 at the protein level were located in the amino acid positions of 557, 499, 604 and 625, respectively, which were not synonym in only the 557 and 499 mutations. In the amino acid position of 557, Alanine was observed in the reference sequence and sequence of the ILC263 as sensitive line while Glycine was observed in the MCC133 as resistant line. At the amino acid position of 499, Asparagine was similarly present in both MCC133 and ILC263 lines, while Aspartate encoded in the reference amino acid sequence. Based on these results, the amino acid of 557 can play a role in the difference in function of resistant and sensitive lines against *Ascochyta rabiei*.

Sequence analysis of this putative protein was performed at SMART and NCBI databases and the results showed that the protein encoded by *GSH118* gene is a Lucien-rich repeat receptor kinases (LRR-RKs) and like its homologue in Arabidopsis with accession number NP_177007 (Theologis et al. 2000), has a Signal Peptide (SP) and several LRR domains at its amino terminus. A transmembrane (TM) domain is present in the protein sequence, and the domains of Serine / Threonine kinases (STKs) and Interleukin-1 Receptor Associated Kinases (IRAKs) are present at its carboxyl end. Protein function likes Kinase activity, protein binding and protein serine/threonine activity have been reported for Arabidopsis homologues of GSH200 (Smakowska-Luzan et al. 2018).

The results of GSH118 protein sequence analysis in Protter web tool well showed that the amino terminus signal peptide is responsible for the secretion of protein out of the cytoplasmic space. Furthermore, the amino acids at the positions of 24–272 are contained leucine-rich domains (LRR) which are located as receptors at the cell surface. The amino acids of 273–298 are responsible for binding the protein to the cytoplasmic membrane, and the carboxyl ends of the protein, including the catalytic domain and several other LRR domains, are located within intracellular space. The LRR domains located in the Extracellular Domains (ECDs) act as a site of interaction and have been shown to be involved in the processes such as microorganisms’ identification. These domains have also been shown to form a network of LRR-based cell surface interaction (CSI^LRR^) which are involved in plant growth and immunity. In fact, the plants use the CSI^LRR^ network to respond to the extracellular signals (Smakowska-Luzan et al. 2018). The transmission of signals received at the cell surface into the cell is affected by intracellular domains. The presence of the kinase domain at the carboxyl end of the GSH118 protein plays an important role in activating intracellular signals. According to the locus of the mutations identified in this domain, the active sits of this domain in the conserved domains presented in the NCBI database were studied with the next accession number (Domain Architecture ID 13746061). The results showed that, there are active sites such as ATP binding conserved residues (α), polypeptide substrate binding site conserved residues (β) and activation loop conserved residues (γ) in these domains and also there are conserved amino acids in each of these sites. More investigation on the position of the considered SNPs showed that SNP18-2147 in the amino acid position of 499 was in the conserved position of the ATP (α) and activation loop (γ) binding sites. At this position of the STKs domain, the amino acid of Aspartate is conserved in the reference proteins of the Arabidopsis (3UIM_A, NP_973956, NP_567053 and CAB86636) while the amino acid of Asparagine has been observed in the sequence of MCC133 and ILC263 lines at the same position. Given the similarity of both amino acids i.e., Aspartate and Asparagine in terms of hydrophilicity and polarity, the only difference is related to the addition of the amino group to Asparagine which can only change its pH. Accordingly, the D499N mutation can be effective in fungal-plant interactions and pH-induced changes.

The *GSH118* gene is a Lucien-rich repeat receptor kinases (LRR-RKs) that encodes a membrane protein with an amino portion outside the cell and a carboxyl portion inside the cell. Due to the presence of ILL domains in the extracellular part of this protein, it has a receptor role and can play an important role in microorganism’s identification. Although the effects of the identified mutations at the protein level were not structurally identified in this study, additional studies are needed. The presence of kinase domains in the intracellular part of the protein can play a key role in signaling pathways activation.

The results of this study showed that using the nucleotide sequences in SNP18, SNP18-2147, SNP18-2491 and SNP18-2554 positions of GSH118 gene, MCC133 (resistant) and ILC263 (sensitive) lines can be distinguished from each other.

Although this SNP was effective in differentiating the Iranian resistant cultivar MCC133 from the susceptible cultivar according to the report of Li et al. (2017), but the experiments performed to investigate the expression of *GSH200* gene in the Iranian resistant cultivar before and after infection by *A. rabiei* did not produce any supportive results. (Data not shown). However, among the three resistant cultivars evaluated in the study of Li et al. (2017), the expression level of *GSH200* gene in one of the resistant cultivars (DICC8218) was not affected like the other two resistant cultivars. Two reasons can be given for the inconsistency between gene expression data and cultivar resistance pattern. First, the candidate gene may not play a role in resistance and second, there may be a variety of defense pathways in different cultivars. For example, in one study, the ethylene receptor (ETR-1) gene was identified as responsible for resistance to AB (Madrid et al. 2013), but in transcriptional profiling studies this gene was not included among the genes that showed differential expression in four chickpea cultivars before and after fungal infection (Coram and Pang 2006). In another study, the expression pattern of 17 candidate genes was evaluated for resistance to AB, but no correlation was found between resistance classification and expression level of genes. It was concluded that the genes that do not have a consistent expression pattern could not be considered as the main resistance gene and it is important to determine the pattern of resistance (Leo et al. 2016). Evaluation of candidate resistance gene analog (RGA) expression in resistant and sensitive plants even though resulted in the identification of four genes with up-regulation in the germination stage and initiation of penetration into the plant's epidermal tissues in the resistant genotype ICC3996 (Zhou et al. 2019). Conversely, our results in the present study showed that none of these genes are present in the 100 Kb region AB4.1 (Li et al. 2017).

Another study that combined data from the methods transcriptome, small RNA and degradome sequencing to discriminate two moderately resistant genotypes (ICCV 05530 and ILC 3279), two susceptible genotypes (C 214 and Pb 7) and one introgression line (BC3F6) showing resistance to AB, 3 and 7 days after inoculation, was also confirmed that many of the distinct genes between parents in the offspring of the backcross do not have a consistent expression pattern (Garg et al. 2019). Accordingly, it is necessary to confirm the linkage between resistance to AB and the expression pattern of candidate genes. However, to determine the linkage of these mutations with the resistance genes, it would be essential to investigate the linkage of alleles within the SNPs positions with the resistance genes in the segregation generations resulting from the crossing of MCC133 (resistant) and ILC263 (sensitive) lines. Additional studies to determine the function of this gene can be effective in gaining more knowledge about the molecular basis of resistance to AB in chickpea.

## Data Availability

All data are presented in figures and tables within this article. Any material used in this study will be available for research purposes upon request.
